# Sequence Variation and Expression of the *Gimap* Gene Family in the BB Rat

**DOI:** 10.1155/2009/835650

**Published:** 2009-05-03

**Authors:** Elizabeth A. Rutledge, Jessica M. Fuller, Brian Van Yserloo, Daniel H. Moralejo, Ruth A. Ettinger, Prashant Gaur, Jana L. Hoehna, Morgan R. Peterson, Richard Jensen, Anne E. Kwitek, Åke Lernmark

**Affiliations:** ^1^Diabetes and Endocrinology Research Center, University of Washington, 815 Mercer Street, Building A, S130, Seattle, WA 98109, USA; ^2^Department of Biological and Chemical Sciences, Salish Kootenai College, 58138 Hwy 93, Pablo, P.O. Box 70, MT 59855, USA; ^3^Department of Clinical Sciences, Clinical Research Center, Lund University, Entrance 72, Building 91:10, 20502 Malmö, Sweden; ^4^Department of Medicine, University of Washington, 1959 N.E. Pacific Street, Seattle, P.O. Box 357710, WA 98195, USA; ^5^Department of Comparative Medicine, University of Washington, 1959 N.E. Pacific Street, Seattle, P.O. Box 357190, WA 98195, USA; ^6^Department of Internal Medicine, University of Iowa, 375 Newton Road, 3111B MERF, Iowa City, IA 52242, USA

## Abstract

Positional cloning of lymphopenia (*lyp*) in the BB rat revealed a frameshift mutation in *Gimap5*, a member of at least seven related GTPase Immune Associated Protein genes located on rat chromosome 4q24. Our aim was to clone and sequence the cDNA of the BB diabetes prone (DP) and diabetes resistant (DR) alleles of all seven *Gimap* genes in the congenic DR.*lyp* rat line with 2 Mb of BB DP DNA introgressed onto the DR genetic background. All (100%) DR.^*lyp*/*lyp*^ rats are lymphopenic and develop type 1 diabetes (T1D) by 84 days of age while DR.^+/+^ rats remain T1D and *lyp* resistant. Among the seven *Gimap* genes, the *Gimap5* frameshift mutation, a mutant allele that produces no protein, had the greatest impact on lymphopenia in the DR.^*lyp*/*lyp*^ rat. *Gimap4* and *Gimap1* each had one amino acid substitution of unlikely significance for lymphopenia. Quantitative RT-PCR analysis showed a reduction in expression of all seven *Gimap* genes in DR.^*lyp*/*lyp*^ spleen and mesenteric lymph nodes when compared to DR.^+/+^. Only four; *Gimap1*, *Gimap4*, *Gimap5*, and *Gimap9* were reduced in thymus. Our data substantiates the *Gimap5* frameshift mutation as the primary defect with only limited contributions to lymphopenia from the remaining *Gimap* genes.

## 1. Introduction

Lymphopenia (*lyp*) is a prerequisite for spontaneous type 1 diabetes (T1D) in the BioBreeding (BB) diabetes prone (DP) rat [1]. Positional cloning of the *lyp* gene revealed a frame shift mutation in *Gimap5* (previously known as *Ian5* or *Ian4L1*). *Gimap5* is a member of at least seven related GTPase Immune Associated Protein (*Gimap*) genes located within 150 Kilobases (Kb) on rat chromosome (RNO) 4 [2, 3]. DR.^*lyp*/*lyp*^ rats, where 2 Mb of DP DNA was introgressed onto the BB diabetes resistant (DR) genetic background, are lymphopenic and 100% develop spontaneous T1D by 84 days of age [4].

The positional cloning and subsequent identification of the *Gimap5* gene on RNO4 were in part established through generation of the DR.*lyp* congenic rat line along with recombination events following our method of marker assisted breeding of DP with F344 rats [2, 4, 5]. Analysis of the *lyp* phenotype in the F344 DNA recombinant rats helped us define the critical *lyp* interval as a region of approximately 33 Kb between D4Rhw6 (76.83 Mb) and IIsnp3 (77.16 Mb) containing *Gimap1*, *Gimap5*, and *Gimap3* (formerly known as *Ian2*, *Ian5*, and *Ian4*, resp.) [2, 4]. *Gimap5* was identified as the *lyp* gene in the BBDP rat through a frameshift mutation and premature truncation of the Gimap5 protein [2, 6] and can be rescued in a P1-derived artificial chromosome (PAC) transgenic rat [7]. However, potential contributions to lymphopenia and/or T1D from the other *Gimap* genes are still unknown. Similarly, how the mechanisms by which reduced *Gimap5* transcript levels and the absence of the Gimap5 protein [2, 7, 8] contribute to lymphopenia and T1D are still being elucidated [9–13].

The predicted structures of the Gimap proteins show common sequences and motifs, such as GTP-binding domains in the N-terminal half, but with differing C-terminal ends [2, 3]. Some C-terminal regions are consistent with transmembrane domains as in the case of Gimap1 and Gimap5, while others, as in Gimap9 and Gimap4, predict coiled coil domains [3, 14]. Both GIMAP4 and GIMAP7 from human Jurkat cells [3] localize to the endoplasmic reticulum and Golgi apparatus while mouse Gimap3 from murine IL-3-dependent 32D myeloid precursor cells was expressed at the outer mitochondrial membrane [15]. Conflicting reports show that GIMAP5, from human primary T cells [10] and from GIMAP5 transfected 293T cells [16], localizes to the centrosome, Golgi apparatus, or endoplasmic reticulum (ER), whereas Gimap5, cloned from Rat2 fibroblasts, localizes to a distinct subcellular fraction that is neither mitochondrial nor ER [11]. Gimap proteins may therefore have similar function, but different subcellular locations. At this time, there is a paucity of information as to the expression of the *Gimap* genes in specific cell types.

The fact that the *Gimap* genes are located together in a tight cluster on RNO4 (and in conserved synteny with many other species), combined with their sequence similarities, suggests the possibility that the proteins carry out similar function. While there is sufficient evidence to support the frameshift mutation in *Gimap5* as the cause of lymphopenia, we could not exclude that either *Gimap1* or *Gimap3* play a role, as they are located within the lymphopenia critical interval between D4Rhw6 and IIsnp3 as well as within the PAC used in the transgenic rescue of lymphopenia [7]. In addition, it is possible that the remaining *Gimap* family members outside the lymphopenia critical interval play a role in T1D development. In order to substantiate the frameshift mutation in *Gimap5* and the subsequent protein null allele as the cause of lymphopenia as well as explore a possible contribution by other *Gimap* family members, we sequenced DR.^+/+^ and DR.^*lyp*/*lyp*^ cDNA from rat thymus. In addition, we examined *Gimap* gene expression across multiple tissues and quantified mRNA expression of all annotated and putative *Gimap* genes in DR.^+/+^ and DR.^*lyp*/*lyp*^ rat thymus, spleen, and mesenteric lymph node (MLN).

## 2. Materials and Methods

### 2.1. DR.*lyp* Congenic Rats

The DR.*lyp* (BBDR.BBDP-(*D4Rhw17-SS99306861*) (*D4Rhw11-D4Rhw10*)/*Rhw*) congenic rat line was derived from animals with two independent recombination events developed from our previously described introgression of the lymphopenia locus by cyclic cross-intercross breeding of BBDP with BBDR rats [17]. The first recombination event was flanked by simple sequence length polymorphism (SSLP) marker D4Rhw11 (76.81 Mb) and the second flanked by SSLP marker D4Rhw10 (77.81 Mb) [4]. Thus, the DP DNA in the DR.*lyp* rat line encompasses the *lyp* critical interval from D4Rhw6 (76.83 Mb) to IIsnp3 (77.16 Mb) [2]. In addition, the DR.*lyp* congenic rat line used in the present study also contains BBDP DNA at D4Rat102 (66.22 Mb) and D4Rat26 (69.18 Mb). The DR.*lyp* congenic rat line is kept in sister-brother breeding and produces Mendelian proportions of the DR.^*lyp*/*lyp*^ (25%), DR.^*lyp*/+^ (50%), and DR.^+/+^ (25%) genotypes. DR.^*lyp*/*lyp*^ are 100% lymphopenic and 100% diabetic.

### 2.2. Housing

Rats were housed in a specific pathogen—free facility at the University of Washington, Seattle, Washington, on a 12-hour light/dark cycle with 24-hour access to food (Harlan Teklad, Madison, Wis, USA) and water. All protocols were approved by the institutional animal use and care committee of the University of Washington, Seattle, Wash, USA. The University of Washington Rodent Health Monitoring Program was used to track infectious agents via a quarterly sentinel monitoring system. Excluded infectious agents are listed at http://depts.washington.edu/compmed/rodenthealth/index.html#excluded.

### 2.3. RNA Isolation

Thymus, spleen, and mesenteric lymph nodes were homogenized from 45–78-day-old DR.*lyp* rats (7 male, 8 female) immediately after dissection in RNA lysis solution (Stratagene, La Jolla, CA or Qiagen, Valencia, Calif, USA) either with a pestle (Kontes, Vineland, NJ, USA) and, if viscous, passed through a 20 gauge needle or a Kinematica Polytron PT 10/35 (Brinkmann, Westbury, NY, USA). Bone marrow was obtained by flushing the femora and tibia with Dulbecco's modified medium (Life Technologies, Grand Island, NY, USA). Nucleated cells were separated with lympholyte-rat (1.094 g/cm^3^, Cedarlane Lab, Ontario, Canada) according to the manufacturer's protocol. Total RNA was isolated using either RNeasy (Qiagen) or Absolutely RNA Miniprep Kit (Stratagene) followed by treatment with DNase. PolyA+ RNA was isolated with Oligotex Direct mRNA Midi/Maxi Kit, (Qiagen). Total RNA was quantitated with RiboGreen (Stratagene).

### 2.4. cDNA Cloning and Sequencing

cDNA synthesis was performed using SuperScript II Reverse Transcriptase (Invitrogen, Carlsbad, Calif, USA) according to the manufacturer's recommendations. PCR products were amplified from thymus cDNA as follows: PCR products were generated by using either Herculase (Stratagene) or Roche Taq polymerase (Roche Diagnostics, Indianapolis, Ind, USA). Reactions with Herculase were 25 *μ*L, consisting of 100 ng cDNA, 0.5 *μ*L of Herculase polymerase, 2.5 *μ*L of the supplied buffer, 0.5 *μ*L of a mix of 10 mM each dNTP, and 2 *μ*L each 5 *μ*M primer. Amplification was carried out in a PTC-200 Peltier Thermal Cycler (Bio-Rad, Hercules, Calif, USA) with the following conditions: 95°C for 3 minutes, 35 cycles of 95°C for 30 seconds, 60°C for 30 seconds, 72°C for 6 minutes, and a final step of 72°C for 7 minutes. Reactions with Roche polymerase were 20 *μ*L, consisting of 100 ng cDNA, 0.1 *μ*L Roche Taq polymerase, 2.0 *μ*L of the supplied buffer, 0.5 *μ*L of a mix of 10 mM each dNTP, and 2 *μ*L each 5 *μ*M primer. Reactions were carried out with the following conditions: 94°C for 3 minutes, 35 cycles of 94°C for 45 seconds, 60°C for 45 seconds, 72°C for 2 minutes, and a final step of 72°C for 7 minutes. PCR products were cloned into pCRII with the TOPO-TA cloning kit (Invitrogen), sequenced using ABI BigDye v3.1 (Applied Biosystems, Foster City, Calif, USA), and analyzed on an ABI 3730XL sequencer (Applied Biosystems) at the University of Washington Biochemistry Sequencing Core in Seattle, WA. 5′ and 3′ RACE (5′ and 3′, Rapid Amplification of cDNA Ends) was carried out with a Marathon cDNA Amplification kit (K1802-1, Clontech, Palo Alto, Calif, USA) using the protocol provided. Plasmids were transformed into XL1Blue (Stratagene) by electroporation of Top10 cells (Invitrogen) according to the protocol supplied with the cells. Plasmids were purified by using GenElute Plasmid Maxiprep Kit (Sigma, St. Louis, Mo, USA) or Plasmid Maxi Kit (Qiagen). GenBank accession numbers of the cloned genes and of RACE products are DQ125335–DQ125353.

### 2.5. Quantitative RT-PCR

RNA was collected from whole tissue, isolated using a Qiaqen RNeasy minikit (Valencia, Calif, USA), and aliquoted to minimize degradation from freezing/thawing. Quantitative real time polymerase chain reaction (qRT-PCR) was performed on anMx4000 Multiplex QPCR System (Stratagene) in duplex reactions with rat cyclophilin (NM_017101) as an internal control. Samples were run in triplicate using 100 ng of total RNA or 5 ng of polyA+ RNA. Twenty-five *μ*L reactions were run using a Brilliant Single-Step qRT-PCR Kit (2.5 *μ*L 10x core RT-PCR buffer, 5.5 mM MgCl_2_, 300 nM each primer, 200 nM each probe, 0.3 mM dNTP, 75 nM passive reference dye, 1.6 units Stratascript RT, 2 units SureStart *Taq* DNA-polymerase). The PCR cycling conditions were as follows: 45°C for 30 minutes, 95°C for 10 minutes, and 40 cycles of 95°C for 30 seconds, 60°C for 1 minute. Probes were positioned in the 3′ regions of the transcripts where there is more variation between the different *Gimap* genes and subjected to BLAST alignment to ensure specificity. The primers and probes used for each gene are listed in Table 1. Primers were obtained from Integrated DNA Technologies (Coralville, Iowa, USA) or Qiagen Operon (Valencia, Calif, USA). Fluorescently labeled probes were obtained from Integrated DNA Technologies. Representative qRT-PCR products for each gene, from each tissue, were run on an agarose gel to check for primer pair binding specificity. Each assay was also optimized and validated with serial dilutions of RNA to produce a standard curve that was then translated into a reaction efficiency, or specificity, of each *Gimap* gene assay. Results from each assay were validated and normalized against cyclophilin. The standard curves, multiplexed with cyclophilin, showed the following reaction efficiencies: *Gimap8*: 90% ± 2 SD, *Gimap7*: 87% ± 4 SD, *Gimap4*: 92% ± 2 SD, *Gimap6*: 94% ± 3 SD, *Gimap9*: 98% ± 7 SD, *Gimap1*: 100% ± 11 SD, *Gimap5*: 88% ± 6 SD, *Lr8*: 93% ± 4 SD, and cyclophilin: 90% ± 6 SD.

### 2.6. Statistical Analysis

Three DR.^+/+^ rats from different litters were used to determine the expression of *Gimap* genes in mesenteric lymph node, thymus, spleen, bone marrow, and kidney. To compare *Gimap* and *Lr8* gene expression across multiple tissues, data was first normalized to cyclophilin then scaled and expressed as a percentage of DR.^+/+^ *Gimap5* mesenteric lymph node (MLN), the highest expressing gene overall. For analysis of *Gimap* expression in DR.^*lyp*/*lyp*^, DR.^*lyp*/+^, and DR.^+/+^ rat thymus, spleen, and mesenteric lymph node, 15 rats (5 rats per genotype) were used from 5 litters consisting of 1 rat genotype from each litter. Mesenteric lymph node data for 1 litter was missing leaving 12 rats from 4 litters for analysis. Comparisons from bone marrow and kidney are not shown due to very low expression and high error in these tissues. Pairwise comparisons of the individual ratios were carried out using linear mixed effects models in S-PLUS (Insightful Corp., Seattle, Wash, USA) with a random intercept for each litter. A conditional *F*-test was implemented to test the significance of terms in the fixed effects models. A two-tail test with a *P*-value < .05 was judged as significant.

### 2.7. Bioinformatics

Predicted protein sequences were aligned using T-coffee (http://www.ch.embnet.org/software/TCoffee.html). Structure and topology of proteins were defined using HMMTOP (http://www.ensim.hu./hmmtop/index.html) or Protein Predict (http://cubic.bioc.columbia.edu/pp/). Subcellular locations were predicted using PSORT (http://psort.nibb.ac.jp/form2.html). The nomenclature used in this paper follows the official names determined by the rat nomenclature committee (Lois J. Maltais, Mouse Genome Database (MGD), Mouse Genome Informatics Web Site, The Jackson Laboratory, Bar Harbor, Maine, http://www.informatics.jax.org/) and is different from previous publications [2, 3, 18, 19].

## 3. Results

### 3.1. cDNA Cloning and Sequencing of *Gimap8, Gimap9, Gimap4, Gimap6*, and *Gimap7* in DR.^+/+^ and DR.^*lyp*/*lyp*^ Rats

**Figure 1:**
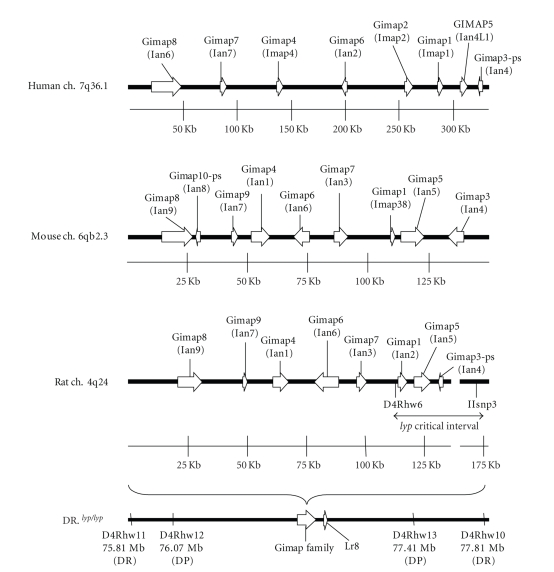
*Expanded map of Gimap interval in human, mouse, and rat.* *Gimap* family orthology in human, mouse, and rat is shown along with an expanded map of the 2 Mb of DP DNA in the congenic DR.*lyp* rat line. The 33 Kb lymphopenia critical interval is indicated between the SSLP markers D4Rhw6 and IIsnp3. *Ian* aliases are in parentheses underneath the corresponding *Gimap* name.

*Gimap8*, *Gimap9, Gimap4, Gimap6*, and *Gimap7* (in the order as they appear on the chromosome) are located outside the lymphopenia critical interval (Figure 1). Sequence analysis of thymus cDNA encoding *Gimap4* showed three single nucleotide polymorphisms (SNPs) at positions 216, 510, and 618, relative to the ATG start site, and two nucleotides deleted at position 922-923 in DR.^*lyp*/*lyp*^ rats as compared to DR.^+/+^ rats (Table 2). The first three base pair substitutions resulted in synonymous amino acid changes in the hypothetical protein sequence, while the deletion resulted in a frameshift mutation in the last three predicted amino acid residues and eliminated the normal stop codon at position 311. 3′ RACE from DR.^*lyp*/*lyp*^ thymus cDNA showed that the reading frame continued for other 21 amino acids before generating a new stop codon (Table 2). This same frameshift mutation was also identified in F344/Rhw (nonlymphopenic) rats, which were used in the positional cloning of lymphopenia (Table 2). One nucleotide substitution was identified in *Gimap7* at position 603, relative to the ATG start site, between DR.^+/+^ and DR.^*lyp*/*lyp*^ that resulted in a synonymous amino acid change in the hypothetical protein sequence (Table 2). No SNPs were found in the coding sequences of *Gimap6*, *Gimap8*, and *Gimap9*.

### 3.2. cDNA Cloning and Sequencing of *Gimap1* and *Gimap3* in DR.^+/+^ and DR.^*lyp*/*lyp*^ Rats

*Gimap1* and *Gimap3* are located inside the lymphopenia critical interval (Figure 1). Sequence analysis of thymic cDNA showed a single SNP in the coding sequence of *Gimap1* at nucleotide position 752, relative to the ATG start site, that produced an amino acid change in DR.^*lyp*/*lyp*^ rats as compared to the DR.^+/+^ (Table 2). The SNP produced a methionine (M) to threonine (T) substitution at amino acid 251, which is located near the C-terminus and is not in any of the predicted GTP binding domains. Sorting intolerant from tolerant (SIFT) analysis (http://blocks.fhcrc.org/sift/SIFT.html) predicted the T substitution to be tolerated at this position and did not predict to affect protein function.

*Gimap3* is not annotated in the rat genome sequence. Genomic sequencing of the putative ortholog of mouse *Gimap3* from base pair positions 76,846,091 to 76,852,162 on RNO 4 (the orthologous DNA interval to mouse *Gimap3*) in DR.^+/+^ and DR.^*lyp*/*lyp*^ rats revealed repetitive single or dinucleotide repeats throughout the region that likely resulted in early termination of the sequencing reactions. As such, no specific PCR products could be generated. Attempts were also made to amplify *Gimap3* from DR.^+/+^ and DR.^*lyp*/*lyp*^ rat thymus cDNA; however, again, no specific PCR products were obtained. Comparative analysis of the Brown Norway (BN/Hsdmcwi) database sequence, available at the University of California Santa Cruz (UCSC; Nov. 2004 assembly), with the mouse *Gimap3* database sequence (UCSC) failed to establish an open reading frame. The multiple repetitive elements added additional difficulty in locating potential exons or transcripts. No rat EST evidence could be found in the region orthologous to mouse *Gimap3* and in human; *GIMAP3* is annotated as a pseudogene. Lastly, no evidence of a *Gimap3* transcript was found in northern blots of DR.^+/+^ and DR.^*lyp*/*lyp*^ or from qRT-PCR of DR.^+/+^ rat thymus or spleen (data not shown). Therefore, *Gimap3* is likely a pseudogene in rat.

### 3.3. Predicted Protein Alignment

**Figure 2:**
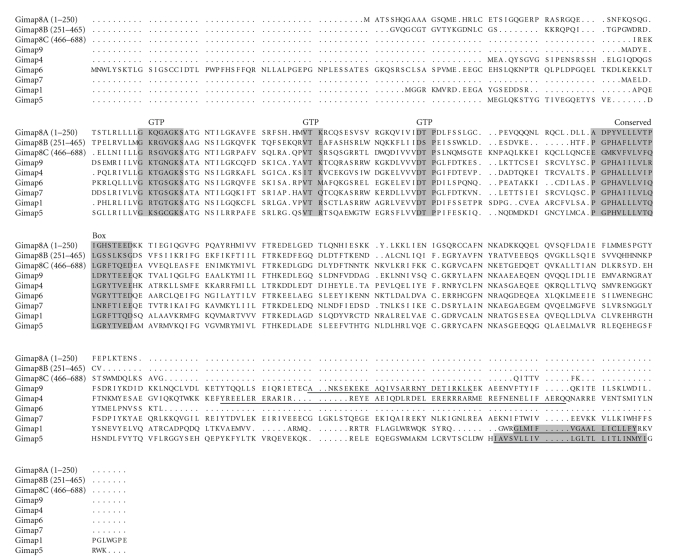
*Alignment of predicted Gimap protein sequences in the DR.^+/+^ rat*. T-coffee predicted protein alignment from cloned cDNA is shown. *Gimap8* is divided into three separate sequences (with the amino acid numbers indicated by each sequence). Shading indicates the GTP binding domain consensus regions (GTP) and the conserved domain (*Conserved Box*). The HMMTOP predicted transmembrane domain sequences for *Gimap1* and *Gimap5* and the coiled coil domains for *Gimap4* and *Gimap9* are underlined.

Alignment of the Gimap family predicted protein sequences in the DR.^+/+^ rat (Figure 2) showed predicted GTP binding domains and conserved box characteristics for all of the Gimap proteins with the most divergent regions located near the C-terminal ends. Gimap1 and Gimap5 are predicted to contain transmembrane domains while Gimap4 and Gimap9 are predicted to contain coiled coil domains. Gimap8, Gimap7, and Gimap6 are predicted to have neither transmembrane nor coiled coil domains. Gimap8 was larger than the other Gimap proteins, containing 688 amino acids and three repeated GTP binding domains (Figure 2).

### 3.4. *Gimap* Expression Pattern across Multiple Tissues

**Figure 3:**
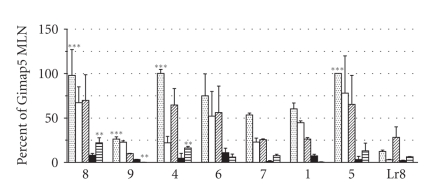
*Tissue specific Gimap expression.* The mean ± standard deviation is shown for DR.^+/+^ (*n* = 3) *Gimap* gene expression. To compare *Gimap* gene expression across multiple tissues, data was first normalized to cyclophilin then scaled and expressed as a percentage of DR.^+/+^ *Gimap5* mesenteric lymph node (MLN), the highest expressing gene overall. Genes appear in the order at which they appear on rat chromosome 4. Tissues appear in the following order per gene: MLN (dots), thymus (white), spleen (hash marks), bone marrow (black), and kidney (stripes). Significance is represented as follows: ∗∗∗ is *P* < .0001 and ∗∗ is *P* < .001.

The relative expression levels of the *Gimap* genes in mesenteric lymph node, thymus, spleen, bone marrow, and kidney were determined in the DR.^+/+^ rat (Figure 3). All of the *Gimap* genes expressed more robustly in the mesenteric lymph nodes, thymus, and spleen as compared to bone marrow and kidney (*P* < .0001). In the mesenteric lymph node, *Gimap4, Gimap5*, and *Gimap8* were expressed significantly higher than *Gimap9* (*P* < .0001) while in kidney, *Gimap4* and *Gimap8* were expressed significantly higher than *Gimap1* and *Gimap9* (*P* < .001) (Figure 3). No significant expression differences were detected between any of the *Gimap* genes (*Gimap8, 9, 4, 6, 7, 1*, and *5*) in thymus, spleen, and bone marrow. Overall, *Lr8,* a gene unassociated with the *Gimap* family but also within the 2 Mb of DP DNA in the congenic DR.^*lyp*/*lyp*^ rat line, expressed predominantly in the spleen, an expression pattern unique relative to the *Gimap* family.

### 3.5. *Gimap* Expression in DR.^*lyp*/*lyp*^ and DR.^+/+^ Thymus, Spleen, and Mesenteric Lymph Node

**Figure 4:**
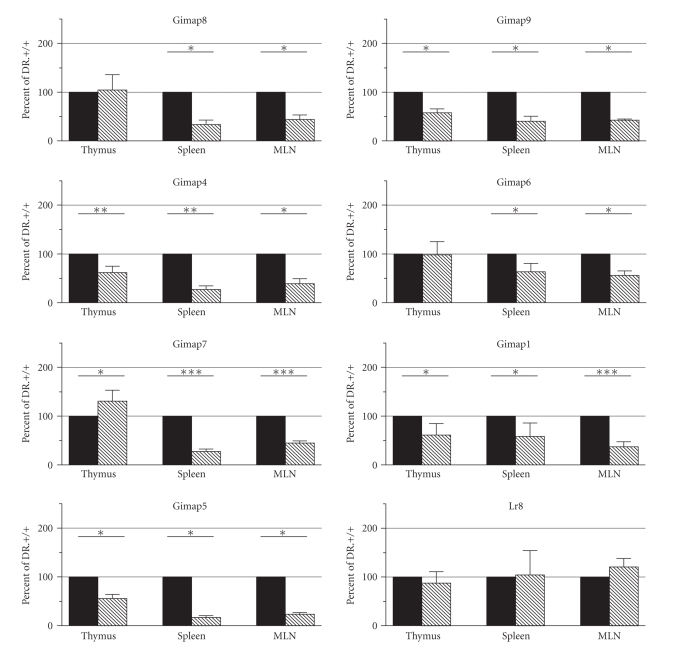
*Gimap gene expression in the DR.^+/+^ and DR.^*lyp*/*lyp*^ rats.* The mean ± standard deviation is shown for DR.^+/+^ and DR.^*lyp*/*lyp*^ rat *Gimap* gene expression in thymus (*n* = 5), spleen (*n* = 5) and mesenteric lymph node (MLN) (*n* = 4) after normalization to cyclophilin. Black columns represent DR.^+/+^ and grey hatched columns represent DR.^*lyp*/*lyp*^. Data is expressed as a percentage of DR.^+/+^. Significant differences are follows; ∗ for *P* < .05, ∗∗ for *P* < .01, ∗∗∗ for *P* < .001. Genes appear in the order at which they appear on rat chromosome 4. The average *Gimap* expression in DR.^+/+^ rat bone marrow and kidney s is shown in Figure 3.

In thymus, expression of *Gimap4, Gimap9, Gimap1*, and *Gimap5* was significantly decreased in DR.^*lyp*/*lyp*^ rats as compared to DR.^+/+^ (Figure 4). In contrast, *Gimap7* expression in thymus was higher in DR.^*lyp*/*lyp*^ as compared to DR.^+/+^ while *Gimap8, Gimap6*, and *Lr8* showed no differential expression. Expression of all of the *Gimap* genes (*8, 9, 4, 6, 7, 1,* and *5*) was reduced in DR.^*lyp*/*lyp*^ rat spleen and mesenteric lymph node as compared to DR.^+/+^ (Figure 4). We observed the same expression pattern whether the data were normalized to cyclophilin or to total RNA (data not shown). Data from bone marrow and kidney is not shown due to the very low expression in these tissues. The low expression observed in these tissues is not due to RNA degradation, but rather to the low mRNA levels relative to cyclophilin levels.

## 4. Discussion

While the frameshift mutation in *Gimap5* is likely necessary and sufficient for lymphopenia, the possibility remains that additional mutation(s) in the *Gimap* family may contribute to the development of lymphopenia, spontaneous T1D, or both in the DR.^*lyp*/*lyp*^ rat. Aside from *Gimap5*, only *Gimap1* and *Gimap3* could potentially play a role in the development of lymphopenia, as they are the only remaining genes located within the 33 Mb interval critical for lymphopenia between D4Rhw6 and IIsnp3. The methionine to threonine base pair substitution at amino acid position 251 in *Gimap1* is not located in any of the predicted GTP binding domains and SIFT analysis predicted that the mutation is not likely to alter normal Gimap1 protein function. Furthermore, the DR amino acid at position 251 is not conserved across species; human GIMAP1 has a valine at this same position [3]. Lastly, no *Gimap3* transcript could be found and no open reading frame could be identified. We suspect that this region of the rat genome does not code for a protein, which is similar to the human GIMAP3 pseudogene [3] and unlike mouse *Gimap3* [15]. Therefore, the sequence analysis of *Gimap1* and *Gimap3* supports the hypothesis that *Gimap5* is the cause of lymphopenia in the DR.^*lyp*/*lyp*^ rat. In addition, the sequence data from all of the remaining *Gimap* family members suggest that these genes are not likely to play a role in the onset of T1D diabetes. We cannot however exclude the possibility that there are mutations outside of the coding regions, such as in transcription factor binding sites or other regulatory sites, that play a role in the regulation of *Gimap* gene expression and/or the onset of T1D in the DR.^*lyp*/*lyp*^ rat.

While we can exclude the involvement of the members of the *Gimap* family proximal to D4Rhw6 (*Gimap8, Gimap9, Gimap4, Gimap6*, and *Gimap7*) in the development of lymphopenia, we cannot exclude that they may play a role in the development of T1D. Coding sequence analysis of these family members revealed that only *Gimap4* had genetic differences, specifically a two-base pair deletion, that would result in a nonsynonymous amino acid change between the nondiabetic DR.^+/+^ and the diabetes susceptible DR.^*lyp*/*lyp*^ (Table 2). Although the effect of this variation is unknown, we did discover this same deletion in the nonlymphopenic, diabetes resistant F344 rat. F344 DNA introgressed through this interval on the DR.^*lyp*/*lyp*^ background protects from onset of T1D [4] suggesting that the deletion mutation in *Gimap4* is not deleterious. In addition, the predicted protein sequences of both human (AK001972) and mouse (NP_778155.2) Gimap4 show that the 23 C-terminal amino acids are similar to those of DR.^*lyp*/*lyp*^ (data not shown). It is therefore unlikely that the *Gimap4* two-base pair deletion mutation in the DR.^*lyp*/*lyp*^ rat is functionally relevant to development of T1D or lymphopenia, rather it is likely an additional natural isoform [20].

All of the *Gimap* genes were predominantly expressed in organs of the immune system: mesenteric lymph node, thymus, and spleen, consistent with previous findings of a role of the *Gimap* gene family in lymphocyte development [21]. Interestingly, there was an overall reduction in expression of all seven *Gimap* genes in DR.^*lyp*/*lyp*^ rat spleen and mesenteric lymph node and four (*Gimap4*, *Gimap9*, *Gimap1*, and *Gimap5*) of the seven genes in DR.^*lyp*/*lyp*^ rat thymus. In contrast, *Lr8,* a gene unrelated to the *Gimap* family located 69 Kb downstream of *Gimap5*, (Figure 1) [22, 23] showed no differential expression between DR.^+/+^ and DR.^*lyp*/*lyp*^. It is not clear why *Gimap5* transcript levels are reduced as the single cytosine residue deletion results in a frameshift mutation and a premature truncation in the protein. One hypothesis is that during protein synthesis, the incomplete (truncated) protein may destabilize the RNA/protein complexes and cause mRNA degradation [24, 25]. Another hypothesis for reduction in DR.^*lyp*/*lyp*^ rat *Gimap* gene transcripts is a difference in organ composition from those of DR.^+/+^ rats. Reduced T cells numbers in DR.^*lyp*/*lyp*^ rats could lead to a difference in cellular composition and may explain the lower observed expression of all of the *Gimap* genes. As the function of Gimap proteins remains rather poorly defined, it would be a useful addendum if in vitro knockdown experiments were designed to test the functional changes that might derive from reduced expression of *Gimap5.* These types of experiments could determine if the mutation is significant in functional terms or if the altered expression is the most critical feature.

The *Gimap* gene family is conserved throughout evolution from plants to humans [3]. Members of the *Gimap* gene family are implicated in a variety of basic cellular processes. These include protection against plant pathogens [26] and okadaic acid and gamma radiation [16]. *Gimap* family members have also been found to be important for T cell development [27], B cell activation [28], B cell malignancy [29], and apoptosis [8, 30]. In addition, *Gimap5* deficient mice have been shown to have impaired maturation and survival of CD4 and CD8 positive T cells [13].

In conclusion, positional cloning of the lymphopenia gene in the spontaneously T1D prone BB DP rat revealed a frameshift mutation in the GTPase Immune Associated Protein *Gimap5* [2, 6]. Our coding sequence analysis of the remaining members of the *Gimap* family revealed that *Gimap4* and *Gimap1* each had coding variation between DR.^+/+^ and DR.^*lyp*/*lyp*^. However, the amino acid differences do not appear to have functional effects substantiating the *Gimap5* frameshift mutation as the cause of lymphopenia. Quantitative real time PCR analysis showed a reduction in expression of all seven *Gimap* genes in DR.^*lyp*/*lyp*^ spleen and mesenteric lymph nodes when compared to DR.^+/+^ while only four, *Gimap1, Gimap4, Gimap5, and Gimap9*, were reduced in thymus. Further understanding of the nature of the *Gimap* family will aid in our goal of characterizing the pathways involved in the development of lymphopenia and T1D.

## Figures and Tables

**Table 1:** Probes and primers used in qRT-PCR.

Primer name	Primer sequence 5′ to 3′
Gimap8-f	CCAGGAGACCCAGGTGAAAG
Gimap8-r	AGTTGAATGCTCATCATAGCTCCTT
Gimap8-p	6FAM-TCTGTTGACAATAGCCAATGATCTCA-BHQ1

Gimap9-f	AGGAACGGCAGAGCCTACTTT
Gimap9-r	CCACTAGACATTGGTTCAGCTTCTTA
Gimap9-p	6FAM-CTGACAGGATATATAAGGACA-MGBQ

Gimap4-f	AACATGCCGTACAGAGCTCACA
Gimap4-r	AGTGGCACCATTAGAAGGCAAA
Gimap4-p	6FAM-CCATGACACACCCACTCCAACAGGG-BHQ1

Gimap6-f	TGGATGCTCTGGATGTTGCA
Gimap6-r	TCCTGCTCATCCCCTTGTG
Gimap6-p	6FAM-TTGTTGAAGCCACAATGGCGTCTCTCA-BHQ1

Gimap7-f	GGACTCAGTGTCAGGCTCCAA
Gimap7-r	CGGGAGGACAGGCTAGCATA
Gimap7-p	6FAM-CTGGATCACACTTGGCGCTCAGCTC-BHQ1

Gimap1-f	AGAGGCGGACCAGGTTCCTA
Gimap1-r	CCTCCAGCCCTGCCTGTAG
Gimap1-p	6FAM-TTCTGCCATCTCCACAGCCCA-BHQ1

Gimap5-f	CATGTTAGGGAAGCTCAGTC
Gimap5-r	GAAGGGTTCTACTGTGTCTCA
Gimap5-p	6FAM-TTTCACTATCATTTGACTCCTGTGCA-BHQ1

Gimap3-f	CCACAGGGAGTGTAGACCTTGAA
Gimap3-r	CTGCTGTTTCCGAATCCAGTTT
Gimap3-p	6FAM-ATCCTCCAGCGTCCAC-MGBQ

Lr8-f	GCCTCTGGTTGTGCCTTCTG
Lr8-r	CCCTGTCCCATCTCATGGAT
Lr8-p	6FAM-CCCACTCCAGCCAAAATTGCCACA-BHQ1

Cyc-f	CACCGTGTTCTTCGACAT
Cyc-r	TTTCTGCTGTCTTTGGAACT
Cyc-p	HEX-CTGCTTCGAGCTGTTTGCAGAC-BHQ1

Probes and primers were designed to bind near the 3′ end of the transcripts. f is forward primer, r is reverse primer, p is probe, 6FAM is 6-carboxyfluorescein, HEX is hexachlorofluorescein, and BHQ1 is black hole quencher 1.

**Table 2:** *Gimap* family thymus cDNA sequencing in DR.^+/+^ and DR.^*lyp*/*lyp*^ rats.

Gene name	Location (bp)	RefSeq identifier	mRNA position	DR.^+/+^	DR.^*lyp*/*lyp*^	F344	A.A Change	Genbank accession #
Gimap8	76738163	NM_001033923	−96	C	T		5′ UTR	DQ125335-36
			−11	T	C		5′ UTR	
Gimap9	76765555	NM_001008398	928	C	T		3′ UTR	DQ125337-38
**D4Rhw2**	**76.77**							
Gimap4	76777679	NM_173153	216	A	G	A	G 72 G (Synonymous change)	DQ125339-40
			510	G	A	G	T 170 T (Synonymous change)	
			618	G	A	G	L 206 L (Synonymous change)	
			922-923	TA	—	—	YLN*	
							308	
							LELIIKAWEIASFIFNQFMRD*	
Gimap6	76794903	NM_001011968	No SNPs					DQ125342-43
Gimap7	76812445	NM_001024328	603	G	A		V 201 V (Synonymous change)	DQ125348-49
**D4Rhw6**	**76.82**							
Gimap1	76829536	NM_001034849	752	T	C		M 251 T	DQ125350-51
Gimap5	76836521	NM_145680	252	C	—		IFESKIQNQDMDKDIGNCY…	DQ125352-53
							85	
							SSSQRSRTKTWTRTLGTAT*	
			523	C	T		L 175 -	
**IIsnp3**	**77.16**							

mRNA position is relative to the ATG start site. UTR is untranslated region.

## References

[1] Markholst H, Eastman S, Wilson D, Andreasen BE, Lernmark Å (1991). Diabetes segregates as a single locus in crosses between inbred BB rats prone or resistant to diabetes. *The Journal of Experimental Medicine*.

[2] MacMurray AJ, Moralejo DH, Kwitek AE (2002). Lymphopenia in the BB rat model of type 1 diabetes is due to a mutation in a novel immune-associated nucleotide (*Ian*)-related gene. *Genome Research*.

[3] Krücken J, Schroetel RMU, Müller IU (2004). Comparative analysis of the human *gimap* gene cluster encoding a novel GTPase family. *Gene*.

[4] Fuller JM, Kwitek AE, Hawkins TJ (2006). Introgression of F344 rat genomic DNA on BB rat chromosome 4 generates diabetes-resistant lymphopenic BB rats. *Diabetes*.

[5] Moralejo DH, Park HA, Speros SJ (2003). Genetic dissection of lymphopenia from autoimmunity by introgression of mutated *Ian5* gene onto the F344 rat. *Journal of Autoimmunity*.

[6] Hornum L, Rmer J, Markholst H (2002). The diabetes-prone BB rat carries a frameshift mutation in *Ian4*, a positional candidate of 
*Iddm1*. *Diabetes*.

[7] Michalkiewicz M, Michalkiewicz T, Ettinger RA (2004). Transgenic rescue demonstrates involvement of the *Ian5* gene in T cell development in the rat. *Physiological Genomics*.

[8] Pandarpurkar M, Wilson-Fritch L, Corvera S (2003). Ian4 is required for mitochondrial integrity and T cell survival. *Proceedings of the National Academy of Sciences of the United States of America*.

[9] Ilangumaran S, Forand-Boulerice M, Bousquet SM (2009). Loss of GIMAP5 (GTPase of immunity-associated nucleotide binding protein 5) impairs calcium signaling in rat T lymphocytes. *Molecular Immunology*.

[10] Dalberg U, Markholst H, Hornum L (2007). Both *Gimap5* and the diabetogenic BBDP allele of *Gimap5* induce apoptosis in T cells. *International Immunology*.

[11] Keita M, Leblanc C, Andrews D, Ramanathan S (2007). GIMAP5 regulates mitochondrial integrity from a distinct subcellular compartment. *Biochemical and Biophysical Research Communications*.

[12] Kupfer R, Lang J, Williams-Skipp C, Nelson M, Bellgrau D, Scheinman RI (2007). Loss of a *gimap/ian* gene leads to activation of NF-*κ*B through a MAPK-dependent pathway. *Molecular Immunology*.

[13] Schulteis RD, Chu H, Dai X (2008). Impaired survival of peripheral T cells, disrupted NK/NKT cell development, and liver failure in mice lacking *Gimap5*. *Blood*.

[14] Stamm O, Krücken J, Schmitt-Wrede H-P, Benten WPM, Wunderlich F (2002). Human ortholog to mouse gene *imap38* encoding an ER-localizable G-protein belongs to a gene family clustered on chromosome 7q32-36. *Gene*.

[15] Dahéron L, Zenz T, Siracusa LD, Brenner C, Calabretta B (2001). Molecular cloning of Ian4: a BCR/ABL-induced gene that encodes an outer membrane mitochondrial protein with GTP-binding activity. *Nucleic Acids Research*.

[16] Sandal T, Aumo L, Hedin L, Gjertsen BT, Døskeland SO (2003). Irod/Ian5: an inhibitor of *γ*-radiation- and okadaic acid-induced apoptosis. *Molecular Biology of the Cell*.

[17] Bieg S, Koike G, Jiang J (1998). Genetic isolation of *iddm 1* on chromosome 4 in the biobreeding (BB) rat. *Mammalian Genome*.

[18] Krücken J, Epe M, Benten WPM, Falkenroth N, Wunderlich F (2005). Malaria-suppressible expression of the anti-apoptotic triple GTPase mGIMAP8. *Journal of Cellular Biochemistry*.

[19] Nitta T, Nasreen M, Seike T (2006). IAN family critically regulates survival and development of T lymphocytes. *PLoS Biology*.

[20] Carter C, Dion C, Schnell S (2007). A natural hypomorphic variant of the apoptosis regulator Gimap4/IAN1. *The Journal of Immunology*.

[21] Filén J-J, FiLén S, Moulder R (2009). Quantitative proteomics reveals GIMAP family proteins 1 and 4 to be differentially regulated during human T helper cell differentiation. *Molecular & Cellular Proteomics*.

[22] Lurton J, Rose TM, Raghu G, Narayanan AS (1999). Isolation of a gene product expressed by a subpopulation of human lung fibroblasts by differential display. *American Journal of Respiratory Cell and Molecular Biology*.

[23] Nakajima H, Takenaka M, Kaimori J-Y (2002). Gene expression profile of renal proximal tubules regulated by proteinuria. *Kidney International*.

[24] Frischmeyer PA, Dietz HC (1999). Nonsense-mediated mRNA decay in health and disease. *Human Molecular Genetics*.

[25] Mendell JT, Sharifi NA, Meyers JL, Martinez-Murillo F, Dietz HC (2004). Nonsense surveillance regulates expression of diverse classes of mammalian transcripts and mutes genomic noise. *Nature Genetics*.

[26] Reuber TL, Ausubel FM (1996). Isolation of arabidopsis genes that differentiate between resistance responses mediated by the RPS2 and RPM1 disease resistance genes. *The Plant Cell*.

[27] Poirier GMC, Anderson G, Huvar A (1999). Immune-associated nucleotide-1 (IAN-1) is a thymic selection marker and defines a novel gene family conserved in plants. *The Journal of Immunology*.

[28] Cambot M, Aresta S, Kahn-Perlès B, de Gunzburg J, Roméo P-H (2002). Human immune associated nucleotide 1: a member of a new guanosine triphosphatase family expressed in resting T and B cells. *Blood*.

[29] Zenz T, Roessner A, Thomas A (2004). hlan5: the human ortholog to the rat lan4/lddm1/lyp is a new member of the Ian family that is overexpressed in B-cell lymphoid malignancies. *Genes & Immunity*.

[30] Lang JA, Kominski D, Bellgrau D, Scheinman RI (2004). Partial activation precedes apoptotic death in T cells harboring an IAN gene mutation. *European Journal of Immunology*.

